# High expression of RRM2 as an independent predictive factor of poor prognosis in patients with lung adenocarcinoma

**DOI:** 10.18632/aging.202292

**Published:** 2020-12-19

**Authors:** Cheng-Yu Jin, Liang Du, A-Han Nuerlan, Xiao-Lei Wang, Yong-Wei Yang, Rui Guo

**Affiliations:** 1 Department of Chest Surgery, People’s Hospital of Xinjiang Uygur Autonomous Region, Urumqi 830001, Xinjiang, China

**Keywords:** RRM2, lung adenocarcinoma, prognostic biomarker, nomogram, gene set enrichment analysis

## Abstract

Ribonucleotide reductase subunit M2 may play a role as a potential prognostic biomarker in several cancers. In this study, we evaluated whether *RRM2* gene expression is associated with the prognosis of patients with lung adenocarcinoma (LUAD) using publicly available data from The Cancer Genome Atlas (TCGA). Wilcoxon signed-rank test and logistic regression were performed to evaluate the association between *RRM2* expression and clinical features in patients with LUAD. Kaplan-Meier and Cox regression methods were used to examine the effect of *RRM2* expression level in the overall survival, and a nomogram was performed to illustrate the correlation between the *RRM2* gene expression and the risk of LUAD. TCGA data set was used for gene set enrichment analysis (GSEA). We also performed a further experiment in vitro to assess the effect of *RRM2* expression on the proliferation and invasive abilities of LUAD cells and its key signaling pathway proteins. Our results revealed that the expression level of *RRM2* in patients with LUAD was much higher than that in normal tissues (*p* = 3.99e-32). High expression of *RRM2* was significantly associated with tumor stage (IV *vs.* I: OR = 3.02, *p* = 0.012) and TNM classification (T2 *vs*. T1: OR = 1.88, *p* = 0.001; N2 *vs*. N0: OR = 2.69, *p* < 0.001). Kaplan-Meier survival analysis showed that high expression of *RRM2* was associated with a worse prognosis of LUAD compared low expression of *RRM2* (*p* = 7.86e-04). Multivariate analysis showed that high *RRM2* expression was an independent factor affecting overall survival (HR = 1.29, *p* < 0.001). The association between *RRM2* gene expression and the risk of LUAD was presented in a nomogram. GSEA revealed that the cell cycle, p53 signaling pathway, DNA replication, small cell lung cancer, apoptosis, and pathways in cancer were differentially enriched in patients with high expression of *RRM2*. *RRM2* over-expression promoted the proliferation and invasive abilities of LUAD cells. RRM2 over-expression increased the activation of Bcl-2 and E-cadherin signaling pathways, and reduced the activation of p53 signaling pathway. In summary, high *RRM2* expression is an independent predictive factor of poor prognosis in patients with lung adenocarcinoma.

## INTRODUCTION

Lung cancer is the leading cause of death globally, approximately 2.1 million new cases and 1.8 million deaths occurred in 2018 [1]. These cases were mainly non-small cell lung cancer (NSCLC), which is divided into different histological categories. Among them, lung adenocarcinoma (LUAD) is the most prevalent subtype [2]. Although multimodal treatment strategies, including immunotherapy, radiotherapy, and non-invasive surgical resection have greatly advanced in recent decades, the outcomes of curing lung cancer remain unsatisfactory and the five-year relative overall survival rate is approximately 18% [3].

Improvements in molecular pathology detection methods and targeted therapies have markedly increased the overall survival of patients with LUAD based on the emerging concept of “precision medicine” in recent years [4]. In precision medicine, key genes driving carcinogenesis can be considered as therapeutic targets. Frequently reported molecules used in pathology detection and as therapeutic targets in adenocarcinoma include epidermal growth factor receptor mutations, echinoderm microtubule-associated protein-like gene, and anaplastic lymphoma kinase gene [5–8]. Although molecularly targeted therapies have shown good clinical results [9–11], curing patients with LUAD remains challenging, particularly because of the development of drug resistance [12, 13]. Therefore, it is necessary to identify more effective indicators for molecular pathology diagnosis and prognosis prediction in patients with LUAD.

Ribonucleotide reductase M2 subunit (RRM2), a rate-limiting enzyme involved in DNA synthesis and damage repair, plays vital roles in many critical cellular processes such as cell proliferation, invasiveness, migration, and senescence [14]. *RRM2* functions as a tumor driver is frequently overexpressed in various malignancies [15, 16]. Lu et al. found that the expression level of *RRM2* is correlated with invasion depth, poorer differentiation, and tumor metastasis in patients with colorectal carcinoma [17]. *RRM2* knockdown attenuated melanoma growth both *in vitro* and *in vivo*, which was correlated with maintenance of senescence-associated cell-cycle arrest [18]. Hsu et al. found that *RRM2* was positively correlated with tumor grade, and patients with early stage NSCLC with *RRM2*-low tumors had better outcomes [19]. These findings indicate *RRM2* may not only function as an oncogene, but also is a promising biomarker for molecular pathology diagnosis and prognosis prediction in patients with LUAD.

This study was conducted to assess the prognostic significance of RRM2 gene expression in LUAD through bioinformatics analysis of the clinical features and survival information from The Cancer Genome Atlas (TCGA). We also performed in vitro experiment to investigate the effect of *RRM2* expression on cell proliferation and invasion of LUAD cells and related key signaling pathways. Our results demonstrated that *RRM2* is useful for predicting the prognosis of patients with LUAD and that high expression of *RRM2* is associated with poor prognosis in these patients.

## RESULTS

### Baseline characteristics of patients

A total of 503 patients with the required clinical features were acquired from TCGA data portal in April 2019. The detailed clinical features are listed in Table 1. Among the 503 participants, 233 were male (46.3%) and 270 were female (53.7%). The median age of all participants was 65 years. In terms of LUAD stage, 268 patients were stage I (54.1%), 120 patients were stage II (24.2%), 81 patients were stage III (16.4%), and 26 patients were stage IV (5.3%). The cancer status included 105 patients with tumors (26.0%) and 301 tumor-free patients (74.0%). The median follow-up of the 503 patients with LUAD was 25.3 months (rang 0–227 months).

**Table 1:** Clinical characteristics of the lung adenocarcinoma patients.

**Clinical characteristics**		**N**	**(%)**
Age (years)	<65	223	44.3
	>=65	280	55.7
Gender	Female	270	53.7
	Male	233	46.3
Race	Asian	9	2.0
	non-Asian	446	98.0
Stage	I	268	54.1
	II	120	24.2
	III	81	16.4
	IV	26	5.3
Smoking status	Current smoker	118	24.1
	Ever smoker	301	61.2
	Never smoker	70	14.7
T classification	T1	171	33.2
	T2	265	53.0
	T3	45	9.0
	T4	19	3.8
M classification	M0	334	93.0
	M1	25	7.0
N classification	N0	323	66.1
	N1	95	19.4
	N2	71	14.5
Location in lung	Central	64	36.8
	Peripheral	110	63.2
Radiation therapy	Yes	13	8.3
	No	144	91.7
New tumor event after initial treatment	Yes	31	16.6
	No	156	83.4
EGFR mutation	Yes	80	29.4
	No	192	70.6
KRAS mutation	Yes	21	35.0
	No	39	65.0
EML4-ALK translocation	Yes	34	14.0
	No	208	86.0
Person neoplasm cancer status	With tumor	106	26.0
	Tumor free	301	74.0
Primary therapy outcomes	SD+PD	19	12.6
	CR+PR	132	87.4

### High RRM2 expression in LUAD

**Figure 1:**
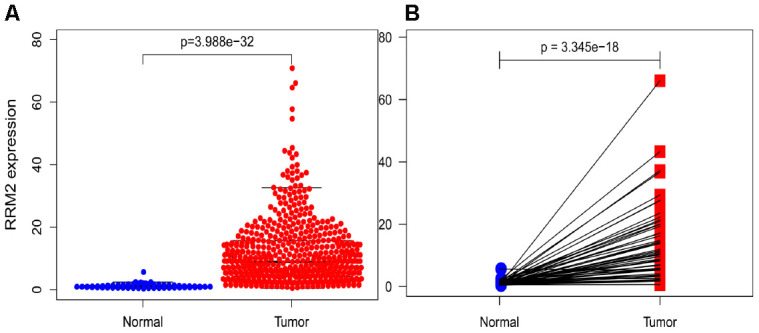
**RRM2 expression in lung adenocarcinoma tissues.** (**A**) RRM2 expression in normal and tumor tissues. (**B**) RRM2 expression in paired tissues.

To assess the status of *RRM2* expression in LUAD patients, we compared the expression level to that in normal lung tissues. The results demonstrated that *RRM2* gene expression level was significantly higher in LUAD tissues (*p* = 3.99e-32) that in normal tissues (Figure 1A). The results were verified in LUAD tissues and paired normal lung tissues (*p* = 3.35e-18) (Figure 1B).

### Correlation between *RRM2* expression and clinical features

**Figure 2:**
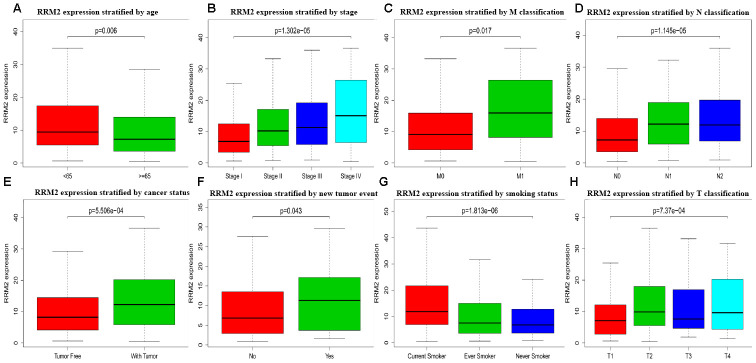
**Box plot evaluating RRM2 expression of patients with lung adenocarcinoma according to different clinical characteristics.** (**A**) Age; (**B**) Clinical stage; (**C**) M classification; (**D**) N classification; (**E**) Cancer status; (**F**) New tumor event; (**G**) Smoking status; (**H**) T classification.

The association identified between *RRM2* expression and clinical features in patients with LUAD is summarized in Table 2. High expression of *RRM2* was significantly correlated with age (*p* = 0.006), clinical stage (*p* = 1.30e-05), T classification (*p* = 7.37e-04), M classification (*p* = 0.017), N classification (*p* = 1.15e-05), cancer status (*p* = 5.51e-04), new tumor event after initial treatment (*p* = 0.043), and smoking status (*p* = 1.81e-06), as shown in Figure 2. However, high expression of *RRM2* was not significantly correlated with other clinical features (Supplementary Figure 1A–1G). Univariate analysis using logistic regression demonstrated that *RRM2* gene expression was a categorical dependent variable associated with poor prognostic clinical features (Table 2). High expression of *RRM2* was significantly associated with clinical stage (IV *vs.* I: OR = 0.012, 95% confidence interval [CI] = 1.31–7.58, *p* = 0.012), T classification (T2 *vs*. T1: OR = 1.88, 95%CI = 1.28–2.79, *p* = 0.001), and cancer status (OR = 1.73, 95%CI = 1.12–2.71, *p* = 0.014).

**Table 2:** Logistic analysis of the association between RRM2 expression and clinical characteristics.

**Clinical characteristics**	**Total (N)**	**RRM2 expression**			
			**OR**	**95%CI**		***p*-Value**
Age (>=65 vs. <65)	503		0.72	0.50-1.03		0.071
Gender (Male vs. Female)	503		1.36	0.96-1.93		0.085
Race (non-Asian vs. Asian)	455		0.99	0.23-4.26		0.995
**Stage**	495					
I			Reference			
II			1.56	1.01-2.40		**0.043**
III			2.18	1.33-3.63		**0.002**
IV			3.02	1.31-7.58		**0.012**
**Smoking status**	489					
Current smoker			Reference			
Ever smoker			0.40	0.25-0.62		**4.66e-05**
Never smoker			0.33	0.18-0.60		**<0.001**
T (T2 vs. T1)	436		1.88	1.28-2.79		**0.001**
M (M1 vs. M0)	359		2.25	0.97-5.65		**0.066**
**N classification**	489					
N0			Reference			
N1			1.78	1.12-2.83		**0.015**
N2			2.69	1.59-4.65		**<0.001**
Location (central vs. peripheral)	174		1.60	0.87-2.97		0.135
Radiation therapy (yes vs. no)	157		2.42	0.75-9.25		0.158
New tumor event after initial treatment (yes vs. no)	187		2.28	1.02-5.37		**0.049**
EGFR mutation (yes vs. no)	272		0.76	0.45-1.28		0.306
KRAS mutation (yes vs. no)	62		0.50	0.17-1.40		0.191
EML4-ALK translocation (yes vs. no)	242		1.16	0.56-2.41		0.692
Person neoplasm cancer status (with tumor vs tumor free)	407		1.73	1.12-2.71		**0.014**
Primary therapy outcomes (SD+PD vs. CR+PR)	151		1.44	0.55-3.94		0.463

Abbreviations: OR, odds ratio; CI, confidence interval.

Bold values indicate P<0.05.

### High expression of *RRM2* is an independent risk factor for overall survival

**Figure 3:**
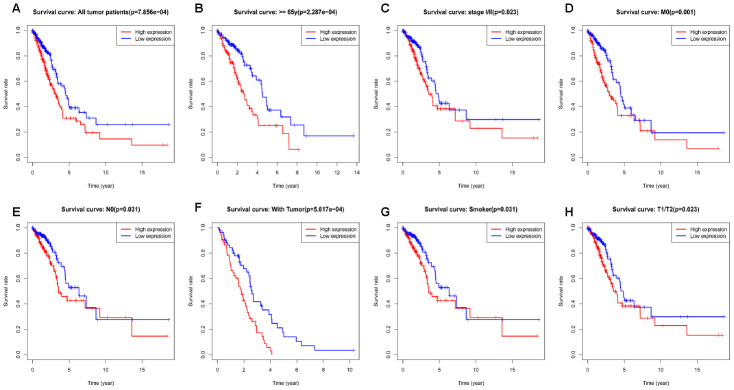
**Kaplan-Meier curve for overall survival in lung adenocarcinoma.** (**A**) Kaplan-Meier curve for RRM2 in all tumor patients; (**B**–**H**) Subgroup analysis for age greater than 65 years, stage I/II, M0, N0, with tumor, smoker, and T1/T2.

**Figure 4:**
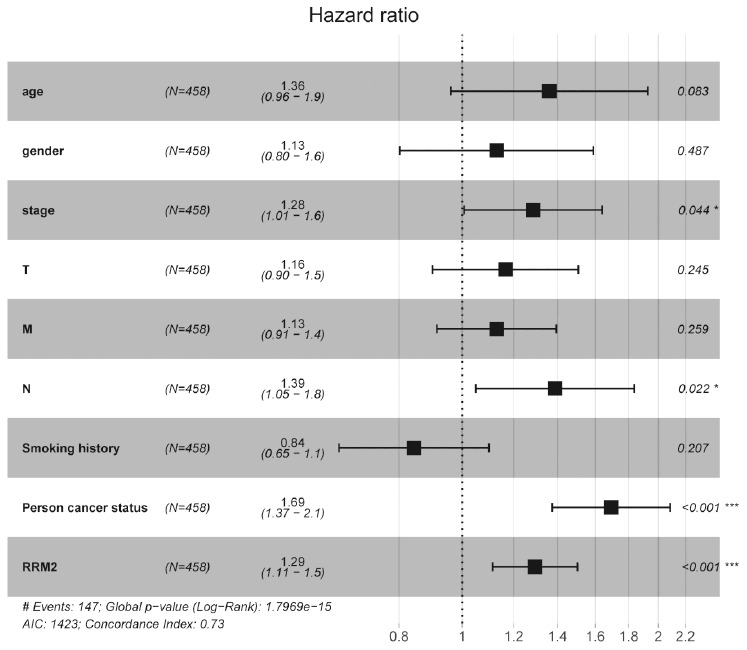
**Forest plot of the multivariate Cox regression analysis in lung adenocarcinoma.**

Kaplan-Meier survival analysis showed that high *RRM2* expression was associated with poor prognosis (*p* = 7.86e-04), as shown in Figure 3A. Subgroup analysis by different clinical features demonstrated that high *RRM2* expression was significantly associated with poor prognosis in LUAD cases more than 65 years old (*p* = 2.287e-04), clinical stage I/II (*p* = 0.023), M0 (*p* = 0.001), N0 (*p* = 0.031), with tumor (*p* = 5.617e-04), smoker (*p* = 0.031), and T1/T2 (*p* = 0.023), as shown in Figure 3B–3H. Univariate Cox analysis demonstrated that high *RRM2* expression was significantly correlated with poor overall survival (hazard ratio [HR] = 1.34, 95%CI = 1.16–1.54, *p* = 4.86e-05). Multivariate Cox analysis confirmed *RRM2* gene expression was an independent risk factor for overall survival in patients with LUAD (HR = 1.29, 95%CI = 1.11–1.50, *p* < 0.001), as shown in Table 3 and Figure 4.

**Table 3:** Univariate and multivariate Cox regression analyses of clinical characteristics associated with overall survival.

**Clinical characteristics**	**Univariate analysis**				**Multivariate analysis**		
	**HR**	**95%CI**	***p*-Value**		**HR**	**95%CI**	***p*-Value**
Age	1.18	0.86-1.63	0.309		1.36	0.96-1.93	0.083
Gender	1.04	0.76-1.43	0.789		1.13	0.80-1.59	0.487
Race	2.01	0.28-14.35	0.488				
Stage	1.67	1.44-1.93	**1.12e-11**		1.28	1.01-1.64	**0.043**
Smoking status	0.92	0.72-1.19	0.540		0.84	0.65-1.10	0.207
T	1.53	1.25-1.86	**3.12e-05**		1.16	0.90-1.51	0.245
M	0.92	0.77-1.11	0.386		1.13	0.91-1.39	0.259
N	1.72	1.44-2.06	**1.82e-09**		1.39	1.05-1.84	**0.022**
Location	0.95	0.57-1.58	0.829				
Radiation therapy	2.87	1.32-6.23	**0.007**				
New tumor event after initial treatment	2.3	1.31-4.05	**0.003**				
EGFR mutation	1.33	0.82-2.15	0.242				
KRAS mutation	2.21	0.85-5.76	0.103				
EML4-ALK translocation	1.96	1.06-3.63	**0.031**				
Primary therapy outcomes	2.44	0.90-6.59	0.078				
Person neoplasm cancer status	4.74	3.27-6.87	**2.08e-16**		1.69	1.37-2.08	**7.20e-07**
RRM2 expression	1.34	1.16-1.54	**4.86e-05**		1.29	1.11-1.50	**<0.001**

Abbreviations: HR, hazard ratio; CI, confidence interval.

Bold values indicate P<0.05.

### Diagnostic value of *RRM2* expression in LUAD

**Figure 5:**
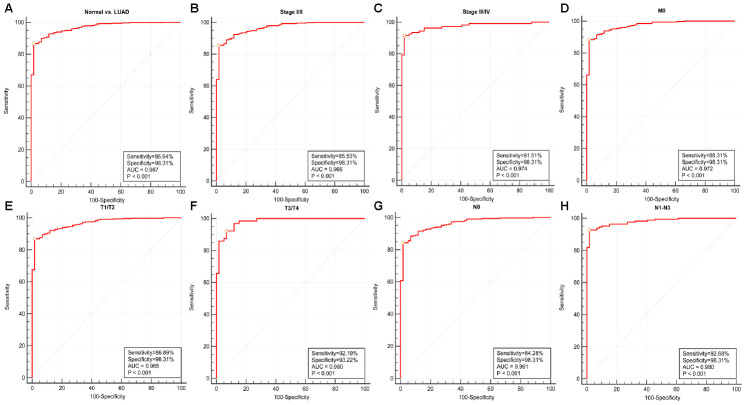
**Diagnostic value of RRM2 expression in lung adenocarcinoma.** (**A**) ROC curve for RRM2 in normal lung tissue and LUAD; (**B**–**H**) Subgroup analysis for stage I/II, stage III/IV, M0, T1/T2, T3/T4, N0, N1-N3.

**Figure 6:**
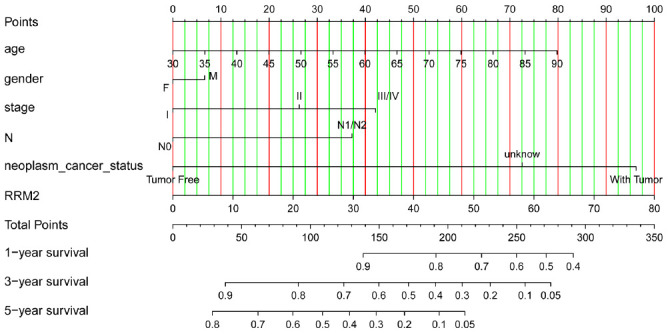
**Nomogram for predicting probability of patients with 1-, 3- and 5-year overall survival.** For risk estimation, identify the status for each clinical factors and expression value of RRM2, draw a line straight upwards to the Points axis to see the points a single factor yields. Repeat until scores for all factors are decided. Sum the points and locate the summed point on the Total Points axis. Then 1-, 3- and 5year related survival probabilities were obtained by draw a line straight down to the Risk axis.

We conducted ROC curve analysis of *RRM2* gene expression data to evaluate the diagnostic value of this gene. The area was 0.967, which indicated high diagnostic value, as shown in Figure 5A. Subgroup analysis demonstrated the diagnostic value of *RRM2* gene expression in different clinical features of LUAD, with AUC values of 0.966 for stage I/II, 0.974 for stage III/IV, 0.972 for M0, 0.961 for N0, 0.980 for N1-N3, 0.965 for T1/T2, and 0.980 for T3/T4 (Figure 5B–H). Therefore, a nomogram was constructed to predict the 1-, 3-, and 5-year survival probability of patients by combining the expression level of *RRM2* with clinical variables (Figure 6).

### GSEA identifies a *RRM2*-related signaling pathway

**Figure 7:**
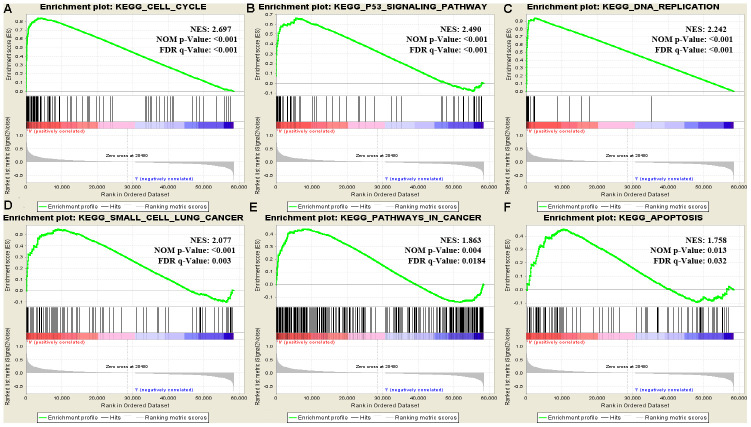
**Enrichment plots from GSEA.** Gene set enrichment plots of (**A**) cell cycle, (**B**) p53 signaling pathway, (**C**) DNA replication, (**D**) small cell lung cancer, (**E**) pathways in cancer, and (**F**) apoptosis in lung adenocarcinoma cases with high RRM2 expression.

We performed GSEA using the low- and high-*RRM2* expression data sets to identify signaling pathways that are differentially activated in LUAD. GSEA showed a large difference (false discovery rate < 5%, nominal *p*-value < 25%) MSigDB collection enrichment (c2.cp.kegg.v6.2.symbols.gmt) (Table 4). Gene sets related to cell cycle, p53 signaling pathway, DNA replication, small cell lung cancer, apoptosis, and pathway in cancer showed differential enrichment in the high *RRM2* gene expression phenotype (Figure 7).

**Table 4:** Gene sets enriched in the high RRM2 expression phenotype.

**Gene set name**	**SIZE**	**ES**	**NES**	**NOM *p*-Val**	**FDR *q*-Val**
KEGG_CELL_CYCLE	124	0.839	2.697	<0.001	<0.001
KEGG_P53_SIGNALING_PATHWAY	66	0.666	2.49	<0.001	<0.001
KEGG_DNA_REPLICATION	36	0.942	2.242	<0.001	1.56e-04
KEGG_SMALL_CELL_LUNG_CANCER	84	0.553	2.077	<0.001	0.003
KEGG_APOPTOSIS	86	0.454	1.758	0.013	0.032
KEGG_PATHWAYS_IN_CANCER	324	0.44	1.863	0.004	0.018

Abbreviations: ES, enrichment score; NES, normalized enrichment score; NOM, nominal; FDR, false discovery rate.

Gene sets with NOM P-value <0.05 and FDR q-value <0.25 were considered as significantly enriched.

### Validation using independent external database

**Figure 8:**
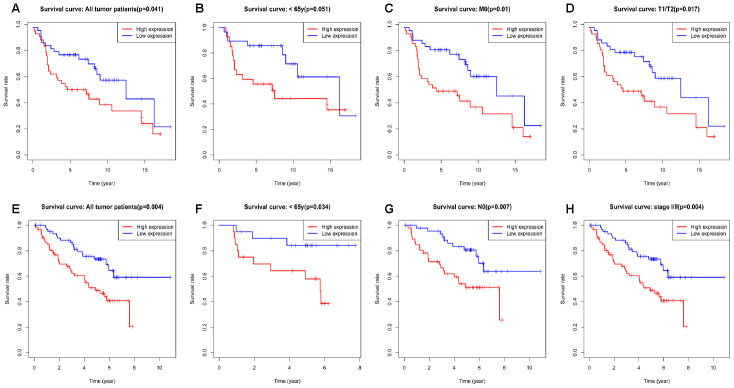
****Kaplan-Meier curve for overall survival in lung adenocarcinoma in the validation datasets GSE30219 (**A**–**D**) and GSE50081 (**E**–**H**).

**Figure 9:**
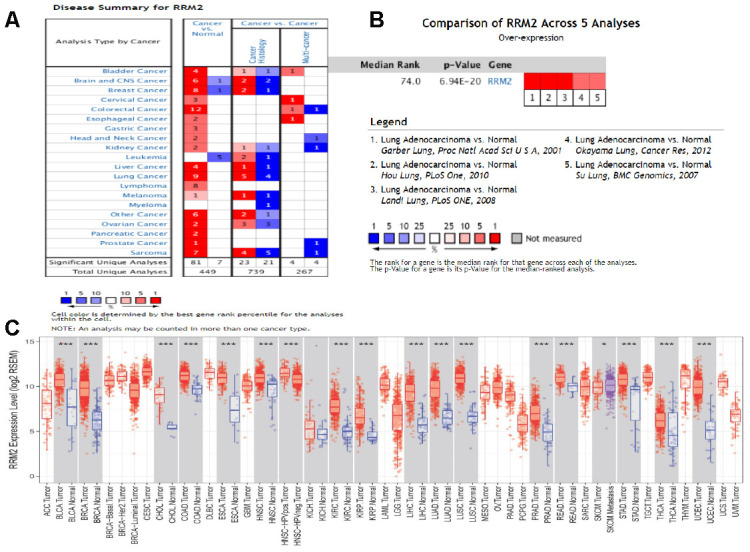
**Expression analysis of RRM2 by Oncomine and TIMER databases.** (**A**) Expression of RRM2 in different types of human cancers in the Oncomine database; (**B**) RRM2 is over-expression (red) in lung adenocarcinoma by Oncomine meta-analysis comparing with normal tissue; (**C**) Expression of RRM2 in different types of human cancers in the TIMER database.

We further validated the repeatability and portability of *RRM2* expression in the prognostic effect of LUAD patients by using two other independent external datasets, including GSE30219 and GSE50081. In the validation set GSE30219, Kaplan-Meier survival analysis revealed that patients with increased *RRM2* expression had shorter OS (Figure 8A–8D). The prognostic value of *RRM2* was also demonstrated in patients with LUAD using GSE50081 (Figure 8E–8H). Meanwhile, the diagnostic value of *RRM2* was also demonstrated in patients with LUAD using GSE30219 (Supplementary Figure 1H). Consistent with these results, *RRM2* was found to be significantly over-expressed in LUAD by using five distinct LUAD datasets (Garber Lung, Hou Lung, Landi Lung, Okayama Lung, and Su Lung) via pooled analysis in the Oncomine database [20–24], and significant over-expression was also found in the TIMER database (Figure 9A–9C). Interestingly, high *RRM2* gene expression is frequently detected in numerous human solid tumors, such as lung cancer, colorectal cancer, breast cancer, bladder cancer, and others (Figure 9A, 9C).

### RRM2 over-expression in LUAD tissues and cell lines was shown by qRT-PCR

**Figure 10:**
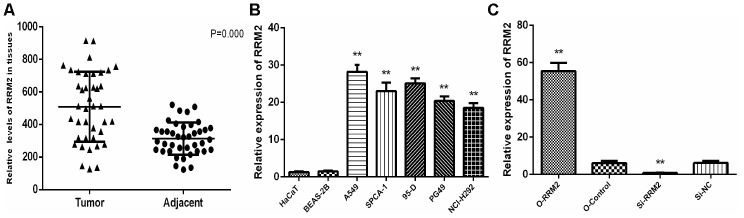
**RRM2 expression in human LUAD tissues and cell lines.** (**A**) Expression of RRM2 in 40 LUAD tissues and corresponding non-cancerous tissues were detected by qRT-PCR; (**B**) RRM2 expression levels in A549, SPCA-1, 95-D, PG-49, BEAS-2B, HaCaT, and NCI-H292 by qRT-PCR; (**C**) RRM2 expression in A549 cells transfected with si-RRM2, O-RRM2, si-NC, and O-Control were confirmed by qRT-PCR. *indicates a *p*-value < 0.05, and **indicates a *p*-value < 0.01.

We examined the expression of RRM2 by qRT-PCR in 40 tumor tissue samples and corresponding non-cancerous tissue samples from LUAD patients. In result, qRT-PCR showed that RRM2 expression was significantly higher in LUAD tissues compared to adjacent non-cancerous tissues (Figure 10A). We also examined the expression of RRM2 in five lung cancer cell lines (A549, SPCA-1, 95-D, PG-49, and NCI-H292), and the results showed that the expression level of RRM2 was significantly higher than that in the normal cell lines (BEAS-2B and HaCaT) (Figure 10B). In particular, the expression level of RRM2 in A549 lung cancer cell line was higher as compared to other lung cancer cell lines, A549 cells thus were selected for the subsequent experiments.

### RRM2 promoted A549 cells proliferation and invasion

**Figure 11:**
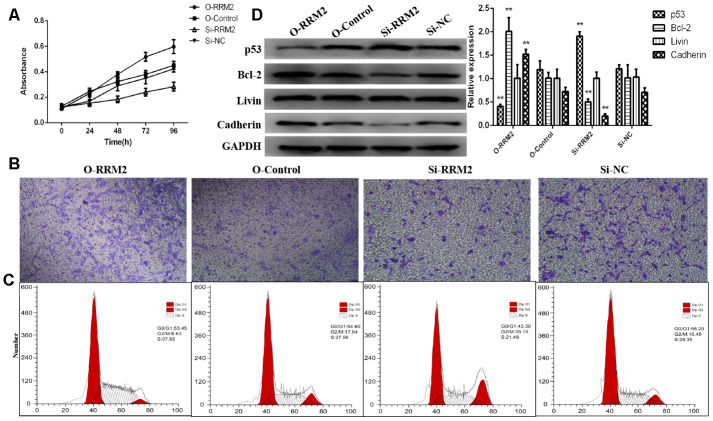
**RRM2 promoted cell proliferation and invasion and regulated cell cycle progression of lung cancer cells.** (**A**) CCK-8 proliferation curve of A549 cells transfected with si-RRM2, O-RRM2, si-NC, and O-Control; (**B**) Transwell assays were performed to evaluate the role of RRM2 on cell invasion; (**C**) The cell cycle analyzed of A549 cells by flow cytometry; (**D**) RRM2-associated signaling pathways were determined by western blot assay. *indicates a *p*-value < 0.05, and **indicates a *p*-value < 0.01.

To explore the role of RRM2 on cell proliferation and invasion, siRNA targeting RRM2 and non-silencing RNA sequences were transfected into A549 cell line, and the efficiency of over-expression and knockdown were validated by qRT-PCR (Figure 10C). In result, qRT-PCR showed that RRM2 expression was markedly lower after transfecting si-RRM2. CCK-8 was used to explore the role of RRM2 on cell proliferation, and the results showed that RRM2 over-expression in A549 cells promoted proliferative activity, whereas RRM2 knockdown markedly inhibited the proliferation ability of A549 cells (Figure 11A). Transwell assays were then employed to evaluate the role of RRM2 on cell invasion, and the results suggest that RRM2 over-expression markedly promoted A549 cells invasive activity (Figure 11B). Cell cycle was further determined by flow cytometry in attempt to explain the RRM2-mediated promotion of proliferative activity. A549 cells revealed G2/M cell cycle arrest after RRM2 knockdown (si-RRM2) (Figure 11C). To verify the underlying mechanism, the relevant signaling pathway proteins, p53, Bcl-2, Livin, and E-cadherin were determined by western blot assay. The results demonstrated RRM2 over-expression in A549 cells increased expression of Bcl-2/E-cadherin and decreased expression of p53, whereas RRM2 knockdown (si-RRM2) markedly decreased the expression of Bcl-2/E-cadherin and increased the expression of p53 (Figure 11D).

## DISCUSSION

Ribonucleotide reductase, an enzyme involved in the cell cycle, has two components identified in humans, the *RRM1* the regulatory subunit and *RRM2* the catalytic subunit, which are vital for DNA synthesis and damage repair [14, 25]. RRM2B is one small subunit of *RRM2*, which is induced by p53 and plays an important role in repairing DNA damage [26]. Maintenance of mitochondrial DNA is also necessary [27]. Ribonucleotide reductase plays an important role in regulating the sizes of dNTP pools, which is required for correct DNA replication [27]. Changes in the sizes of dNTP pools or disruption in their balance will lead to increased mutation rates [27, 28]. Ribonucleotide reductase high expression is a characteristic of many solid tumors, and a series of mechanistically distinct ribonucleotide reductase inhibitors serve as effective agents for cancer treatment [29–31]. As the rate-limiting ribonucleotide reductase enzyme, the *RRM2* level controls the cell cycle-dependent activity of ribonucleotide. Suppression of *RRM2* expression sensitizes cancer cells to both ribonucleotide reductase inhibitors and cisplatin [29, 32]. Overexpression of *RRM2* is associated with poorer prognosis in multiple carcinomas [19, 33]. For instance, patients with cervical cancer with positive *RRM2* expression showed a higher recurrent rate and lower survival rate than those with negative *RRM2* expression [34]. Additionally, *RRM2* dysregulation is related to chemoresistance during cancer treatment. *RRM2* downregulation can overturn AKT-induced tamoxifen resistance and increase therapeutic efficacy in breast carcinoma [35].

*RRM2* gene expression and functions in carcinoma have been recently reported [36–38]. However, the significance of its expression in prognosis in patients with LUAD is largely unclear. In the current study, we analyzed the *RRM2* expression profile in numerous human solid tumors via using Oncomine database. the results demonstrated *RRM2* gene expression was higher in lung cancer, colorectal cancer, breast cancer, bladder cancer, sarcoma cancer, liver cancer, and others than in their matched adjacent normal tissues. *RRM2* gene expression and its potential prognostic impact on patients with LUAD have not been evaluated. In 2015, Mah et al. explored the expression level of *RRM2* in an NSCLC stratification subgroup based on gender and smoking status to predict survival outcomes. the results revealed high *RRM2* expression was associated with primarily women and non-smoking patients [39]. Toffalorio et al. investigated the expression levels of seven genes involved in gemcitabine metabolism in advanced NSCLC, including the *RRM2* gene; the results revealed *RRM2* gene expression was not associated with patients with advanced NSCLC treated with gemcitabine [40]. This is the first comprehensive study to evaluate *RRM2* gene expression in the prognosis of patients with LUAD. In the present study, we evaluated the *RRM2* gene expression profile via bioinformatic analysis using TCGA database. With respect to matched normal lung tissues, *RRM2* gene expression levels were significantly higher in patients with LUAD and the result has been confirmed by qRT-PCR. Kaplan-Meier survival analysis revealed that patients with *RRM2* high expression had shorter OS (*p* < 0.05). This finding was validated in other independent external datasets (all *p* < 0.05). Multivariate Cox analysis further confirmed that *RRM2* high expression was an independent risk factor for OS in patients with LUAD; other clinicopathologic features were also associated with worse prognosis in LUAD, including advanced stage, lymph nodes metastases, with tumor, as suggested by the forest plot. ROC analysis also verified the diagnostic value. At present, a predictive nomogram for LUAD by combining the expression value of *RRM2* with clinical variables has not been reported. Therefore, we constructed a prognostic nomogram by integrating clinical factors and gene expression via TCGA dataset to enable clinicians to predict the risk of individual patient death and guide patient assessment and therapeutic decision-making.

We found that the high *RRM2* expression phenotype was associated with the cell cycle, p53 signaling pathway, DNA replication, small cell lung cancer, apoptosis, and pathways in cancer by GSEA. DNA replication ensures that cellular genetic information is accurately copied and correctly transmitted to offspring cells [41, 42]. However, DNA replication is prone to interference and damage under various pressures in the body, leading to stagnant of DNA replication, affecting genome stability and even inducing apoptosis, necrosis, and carcinogenesis. p53 is a transcription factor that specifically binds to DNA [43]. It can regulate specific gene expression and induce cell cycle suspension, DNA repair, and apoptosis. Pathway enrichment analysis suggested that *RRM2* affects the pathogenesis of proliferation and invasion in lung carcinoma through the above pathways. Han et al. found that *RRM2-c2orf48* over-expression induced epithelial-mesenchymal transition in nasopharyngeal carcinoma cells results in the down-regulation of E-cadherin and promoted nasopharyngeal carcinoma cell migration and invasion [44]. Li et al. demonstrated that *RRM2* is over-expressed in glioblastoma and this over-expression can promote the proliferation, migrative, and invasive abilities, whereas suppress cell apoptosis of glioblastoma cells both in vivo and in vitro [45]. Shah et al. demonstrated that nuclearfactor-kappaB (NF-κB), MAPK/JNK, and EGFR are the major signaling pathways that are affected by the over-expression of *RRM2* and this overexpression increased gene expression of *EGFR*, *Bcl*-2, and *MMP9* and decreased gene expression of *p53*, *CDKN1A*, and *CDKN2A* [46]. Furthermore, *RRM2* over-expression promoted the proliferative activity, migratory and invasive capabilities of breast cancer cells. Several studies have also found *RRM2* protein interact with key cell cycle genes and signaling pathway proteins, such as p53, PI3K, hypoxia inducible factor-1α, Bax, and cyclin D1, to regulate tumor cell proliferation, migratory, and invasive abilities and cell cycle progression [45–48]. Our results, partly in line with the findings in the above studies, showed that *RRM2* promoted lung cancer cell proliferation and invasion and cell cycle progression. However, such mechanisms require further investigation.

In summary, we showed that *RRM2* is upregulated in LUAD, and high *RRM2* expression was correlated with clinical progression and considered as an independent risk factor for OS in patients with LUAD. We also found that *RRM2* promotes the proliferative activity and invasive capabilities of lung cancer cells; suggesting that *RRM2* play a crucial role in tumor initiation, development and malignant behavior and may be used as biomarker in the diagnosis and prognosis of patients with lung adenocarcinoma. Moreover, the p53 signaling pathway, cell cycle, DNA replication, small cell lung cancer, apoptosis, and pathways in cancer may be pivotal pathways regulated by *RRM2* in LUAD. *RRM2* expression may be an important prognostic factor in patients with LUAD.

## MATERIALS AND METHODS

### Patient data sets

mRNA expression data (535 samples, Workflow Type: HTSeq-FPKM) and clinical information were downloaded from TCGA database (https://cancergenome.nih.gov). The following samples were excluded: (1) “0” gene expression value and (2) insufficient survival information. A total of 503 patients with LUAD with the corresponding clinical features were enrolled in this study.

### Gene set enrichment analysis (GSEA)

GSEA is a computational method determines whether a previously defined set of genes have significant statistical and concordant differences in two biological states [49]. GSEA was performed to identify all genes found to be correlated with *RRM2* gene expression in recent studies and to examine the significant survival differences observed between the high- and low-*RRM2* groups. For each analysis, gene set permutation was performed 1000 times. Gene sets with a normal p-vale less than 5% and false discovery rate less than 25% were considered as significantly enriched.

### Sample collection

LUAD and adjacent tissues were collected from 40 patients immediately stored in liquid nitrogen, and preserved at -80° C. The use of all samples conformed to the Declaration of Helsinki. The present study was approved by the Ethics Committee of People’s Hospital of Xinjiang Uygur Autonomous Region

### Cell culture and transfection

Human normal lung epithelial cell line (BEAS-2B), human immortalized keratinocytes cell line (HaCaT; normal control), and human lung cancer cell lines (A549, SPCA-1, 95-D, and NCI-H292) were obtained from the cell bank of the Chinese Academy of Sciences in Shanghai (Shanghai, China), and PG-49 was obtained from American Tissue Culture Collection (ATCC; Manassas, VA, USA). All cells were cultured in Dulbecco's Modified Eagle's medium (DMEM; Gibco, Gran Island, NY, USA) supplemented with 10% fetal bovine serum (FBS; Gibco) and antibiotics (100 units/ml penicillin and 100 ug/ml streptomycin; Gibco), except SPCA-1, PG49, and HaCaT, which were cultured in Roswell Park Memorial Institute-1640 (RPMI-1640; Gibco). All cells were cultured at 37° C, 5% CO2 in a humidified atmosphere incubator.

The lentiviral vector containing RRM2 small interfering RNA (siRNA) was constructed by GeneChem Co., Ltd (Shanghai, China). siRNA targeting RRM2 (NM_001034) sequences was 5'-GGAGCGAUUUAGCCAAGAAGU-3'. Four groups were constructed as follows: si-RRM2 transfected group (si-RRM2), RRM2 overexpressed group (O-RRM2), an empty lentiviral vector was regarded as a negative control (si-NC), and un-transfected group (O-Control). Transfection was performed as described previously [50]. The efficiency of over-expression and knockdown were validated by qRT-PCR. Finally, the transfected A549 lung cancer cells were collected for subsequent experiments.

### Quantitative real-time polymerase chain reaction (PCR) of tissues and cell lines

Total RNA was extracted from cell lines or tissue specimens using TRIzol reagent (Invitrogen, Thermo Fisher Scientific, Shanghai, China) according to the manufacturer’s instructions, and RNA was reversely transcribed into cDNA using Transcription First Strand cDNA synthesis kit (Roche, Basel, Switzerland). Quantitative real-time PCR (qRT-PCR) analyses were quantified with SYBR^®^ Green (Roche, Basel, Switzerland). The relative expression of RRM2 was calculated based on the 2^-ΔΔCt^ method with β-actin as an internal reference. qRT-PCR primers used in the present study were as follows: RRM2 forward primer, 5’-ACGGAGACTCACCAGTTGG-3’; RRM2 reverse primer, 5’-GCACGACGCTGAGGATCAA-3’; β-actin forward primer, 5’-CTCCATCCTGGCCTCGCTGT-3’; β-actin reverse primer, 5’-GCTGTCACCTTCACCGTTCC-3’.

### Cell proliferation and invasion assay

To evaluate the proliferative activity of lung cancer cells, a Cell Counting Kit-8 (CCK-8; Dojindo, Kumamoto, Japan) was performed according to the manufacturer’s instructions. Briefly, A549 cells were plated in 96-well plates (Density: 3 × 10^3^ cells per well) and incubated overnight. Absorbance of 450 nm was measured using a microplate reader (Thermo Fisher Scientific, Shanghai, China) daily for four days. The experiment was independently repeated three times. Transwell chamber assays were performed to assess the invasive ability of lung cancer cells according to the manufacturer’s instructions.

### Western blot assay and antibody

RIPA lysis buffer (Thermo Fisher Scientific, Shanghai, China) containing protease and phosphatase inhibitors was used to lyse lung cancer cells. Protein lysates were separated by SDS-PAGE gels (Thermo Fisher Scientific, Shanghai, China), blotted onto PVDF membrane (Roche, Basel, Switzerland) for analysis and incubated at 4° C for overnight with the following primary antibodies: anti-p53 antibody (1:3000 dilution; #10442-A-AP, Sanying Biotechnology, Wuhan, China), anti-Bcl-2 antibody (1:1000 dilution; #ab32124, Abcam, Cambridge, MA, USA), anti-Livin antibody (1:1000 dilution; #ab97350, Abcam, Cambridge, MA, USA), anti-E-Cadherin antibody (1:2000 dilution; #20874-1-AP, Sanying Biotechnology, Wuhan, China), and anti-GAPDH antibody (1:5000 dilution; #HRP-60004, Sanying Biotechnology, Wuhan, China). The results of the western blot analyses were performed with Image J software.

### Cell cycle assay

A549 cells were plated in six-well plates (Density: 2 × 10^5^ cells per well) and incubated for 12 h. A549 cells were incubated to 80-90% confluency and then washed two times with PBS and fixed with 70% precooled ethanol at 4° C for 12 h. A549 cells were re-washed two times with PBS and cultured with 500 μl propidium iodide (PI)/RNase staining buffer (Cell Signaling Technology, Inc, Danvers, MA, USA) for 30 minutes away from light. The distribution of cells in G0/G1, G2/M, and S phases of the cell cycle was determined by flow cytometry (Beckman Coulter Quanta SC System).

### Statistical analysis

The expression level of the *RRM2* gene in patients with LUAD was evaluated by using box plots. The cut-off value of *RRM2* expression was selected as the median method of gene expression. Wilcoxon signed-rank test and logistic regression were performed to analyze the association between clinical features and *RRM2* expression in LUAD. Kaplan-Meier analysis was performed to compare the overall survival (OS) rate between the high and low *RRM2* gene expression groups using the p-value determined in the log-rank test. A received operating characteristic (ROC) curve was applied to assess the diagnostic value of *RRM2* gene expression, with the area under the ROC curve used as the diagnostic value. Use univariate Cox analysis to screen potential prognostic factors, and multivariate Cox analysis to verify the effect of *RRM2* expression on survival along with other clinical variables. A nomogram was constructed to predict 1-, 3- and 5-year LUAD overall survival by combining the expression value of *RRM*2 with clinical variables. External validation was applied via using two other independent external datasets (GSE30219 and GSE50081). The expression level of *RRM2* in patients with LUAD and normal individuals was further validated in the Oncomine database (https://www.oncomine.org/resource/main.html) and TIMER database (https://cistrome.shinyapps.io/timer/). All statistical analyses were performed using R statistical software (version 3.5.3), SPSS software (version 24.0), or MedCalc software (version 19.1). A *p*-value less than 0.05 is considered as statistically significant.

## Supplementary Material

Supplementary Figure 1
