# Supplementary material for: High expression of RRM2 as an independent predictive factor of poor prognosis in patients with lung adenocarcinoma
Source: Aging (Albany NY). 2020 Dec 19;13(3):3518–35. doi: 10.18632/aging.202292 (PMC7906179; doi:10.18632/aging.202292)
Supplement: Supplementary Figure 1 [file aging-13-202292-s001.pdf]

SUPPLEMENTARY FIGURE

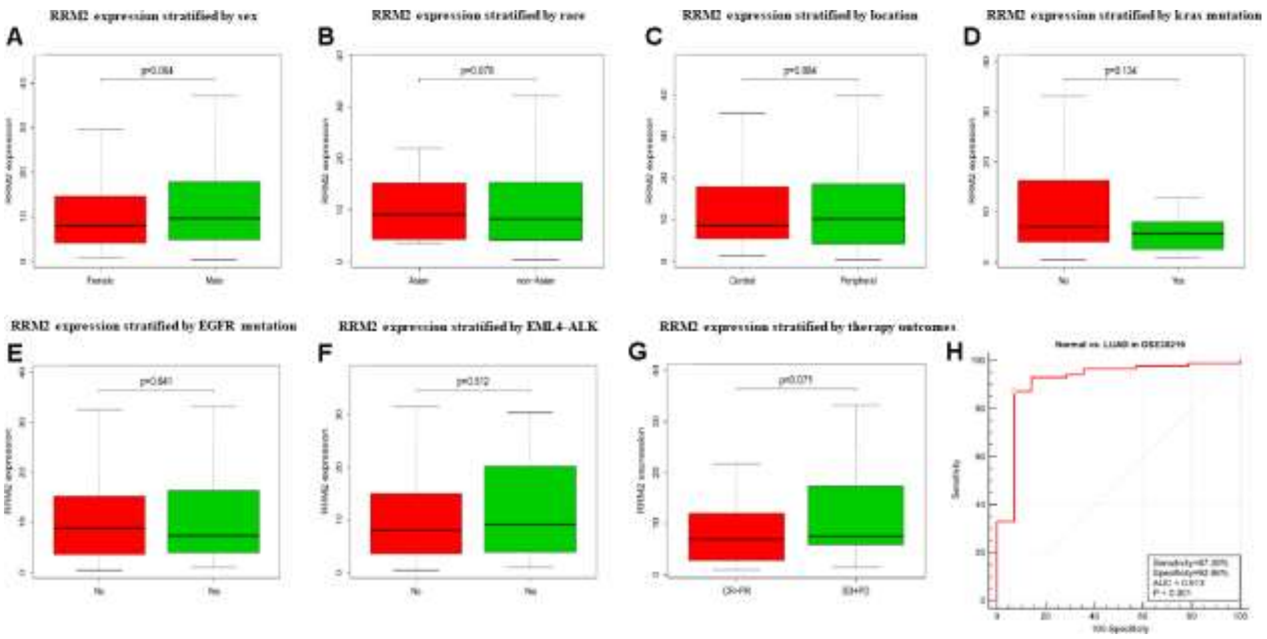

Supplementary Figure 1. Box plot evaluating RRM2 expression of patients with lung adenocarcinoma according to different clinical characteristics. (**A**) Sex; (**B**) Race; (**C**) Location in lung; (**D**) kras mutation status; (**E**) EGFR mutation status; (**F**) EML4-ALK translocation; (**G**) Therapy outcomes and (**H**) Diagnostic value of RRM2 expression in lung adenocarcinoma in GSE30219.
